# Correction: Flexible, micro-porous chitosan–gelatin hydrogel/nanofibrin composite bandages for treating burn wounds

**DOI:** 10.1039/c9ra90033k

**Published:** 2019-05-17

**Authors:** P. T. Sudheesh Kumar, G. Praveen, Mincy Raj, K. P. Chennazhi, R. Jayakumar

**Affiliations:** Amrita Centre for Nanosciences and Molecular Medicine, Amrita Institute of Medical Sciences and Research Centre, Amrita Vishwa Vidyapeetham University Kochi-682041 India rjayakumar@aims.amrita.edu jayakumar77@yahoo.com +91 484 2802020 +91 484 2801234

## Abstract

Correction for ‘Flexible, micro-porous chitosan–gelatin hydrogel/nanofibrin composite bandages for treating burn wounds’ by P. T. Sudheesh Kumar *et al.*, *RSC Adv.*, 2014, **4**, 65081–65087.

The authors regret that there were errors in Fig. 3 and 4 in the original manuscript. The cell viability data of chitosan–gelatin hydrogel/nanofibrin ternary composite bandages using alamar assay against HDF cells in Fig. 3a was inadvertently duplicated as Fig. 3b, which represents the alamar blue cell viability assay against HUVEC cells. The correct Fig. 3 is presented here.

**Fig. 1:**
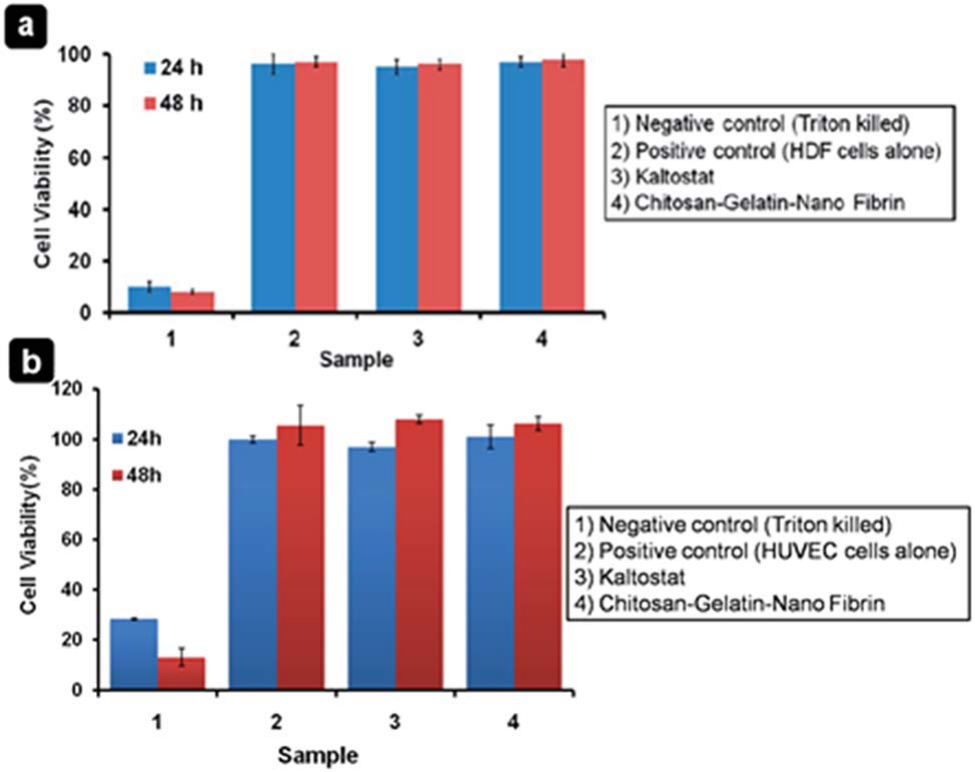
Cell viability of bandages using (a) HDF cells and (b) HUVECs.

The images in the lower panel of Fig. 4 were incorrect in the original manuscript, showing cellular attachment of HUVECs cells on composite bandages without gelatin. The lower panel should have shown DAPI stained images of cellular attachment of HUVEC cells on chitosan-gelatin hydrogel/nanofibrin ternary composite bandages. The corrected Fig. 4 is as presented here.

**Fig. 2:**
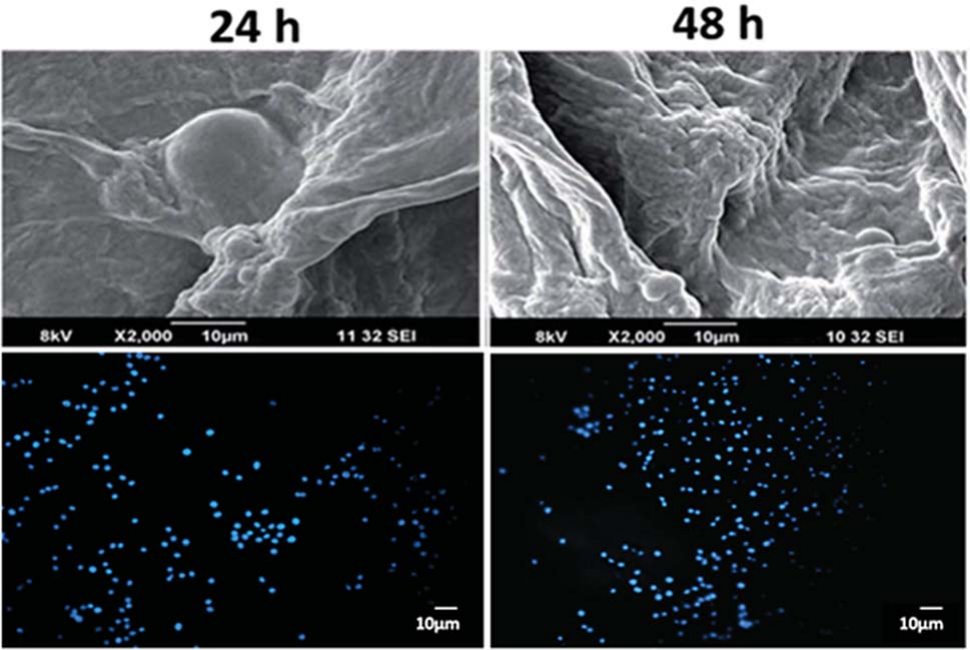
Comparison of cellular attachment of HUVECs on CFGBs as seen by SEM (upper panels) and subsequent cell proliferation visualized through DAPI nuclear staining (lower panels).

The Royal Society of Chemistry apologises for these errors and any consequent inconvenience to authors and readers.

## Supplementary Material

